# Molecular Evidence for High Frequency of Multiple Paternity in a Freshwater Shrimp Species *Caridina ensifera*

**DOI:** 10.1371/journal.pone.0012721

**Published:** 2010-09-14

**Authors:** Gen Hua Yue, Alex Chang

**Affiliations:** 1 Molecular Population Genetics Group, Temasek Life Sciences Laboratory, National University of Singapore, Singapore, Singapore; 2 R and D Department, Qian Hu Fish Farm, Singapore, Singapore; Aarhus University, Denmark

## Abstract

**Background:**

Molecular genetic analyses of parentage provide insights into mating systems. Although there are 22,000 members in Malacostraca, not much has been known about mating systems in Malacostraca. The freshwater shrimp *Caridina ensifera blue,* is a new species belonging to Malacostraca which was discovered recently in Sulawesi, Indonesia. Due to its small body size and low fecundity, this species is an ideal species to study the occurrence and frequency of multiple paternity and to understand of how the low fecundity species persist and evolve.

**Methodology/Principal Findings:**

In this study, we developed four polymorphic microsatellites from *C. ensifera* and applied them to investigate the occurrence and frequency of multiple paternity in 20 *C. ensifera* broods caught from Lake Matano, Sulawesi. By genotyping the mother and all offspring from each brood we discovered multiple paternity in all 20 broods. In most of the 20 broods, fathers contributed skewed numbers of offspring and there was an apparent inverse correlation between reproductive success of sires and their relatedness to mothers.

**Conclusions/Significance:**

Our results in combination with recent reports on multiple paternity in crayfish, crab and lobster species suggests that multiple paternity is common in Malacostraca. Skewed contribution of fathers to the numbers of offspring and inverse correlation between reproductive success of sires and their relatedness to mothers suggest that sperm competition occurred and/or pre- and postcopulatory female choice happen, which may be important for avoiding the occurrence of inbreeding and optimize genetic variation in offspring and for persistence and evolution of low fecundity species.

## Introduction

Inferring of parentage of individuals in natural populations is important in understanding mating behaviour, which is of importance for studying reproductive strategies, sperm competition, cryptic female choice, and evolution [1], [2], [3], [4], [5], [6]. Parentage in natural populations is very difficult to determine by field observations, because usually mating can not be easily observed in the wild. With the advent of polymorphic DNA markers [7] and sophisticated statistic tools [8], parentage in wild populations can now be determined [9]. Mating behaviour and the extent of multiple mating by male and female individuals are important components of life-history traits. It is generally believed that multiple mating posses both advantages and disadvantages. Disadvantages include an increased risk of predation and disease transmission [10]. Benefits of multiple mating include (1) prolonged guarding of vulnerable females by their mate [11]; (2) ensuring fertilization because (a) some males may be sterile, (b) males invest less sperm than females require when they partition their ejaculate among multiple females; or (c) there is a reduction of active sperm in storage organs due to passive loss or sperm mortality over time [12]; (3) avoiding genetic incompatibility [13], inbreeding [14], [15] and genetic defects resulting from stored sperm [16]; and (4) promoting the gain of ‘good genes’ and increasing genetic diversity among offspring [17].

Malacostraca is a large group (22,000 members) including the crabs, shrimps, and lobsters as well as several crustaceans [18], [19]. Although, some studies on mating systems in the class Malacostraca have been conducted [20], [21], they were not as intensive as in vertebrate groups [22] and reptiles [2]. In some species of the class Malacostraca, such as grass shrimp (*Palaemonetes pugio*) the male transfers a spermatophore to the female. The eggs are fertilized externally [23], whereas in some species such as hermit crabs [24] and red swamp crayfish [25], fertilization are internal, mating take place often just after female molts, and eggs often carry in special appendage. In some species (e.g. *Armadillidium vulgare*), females can store sperm for some time [26]. Only in a few species belonging to Malacostraca, such as crayfish *Orconectes placidus* [27], red swamp crayfish *Procambarus clarkii* [28], lobster *Nephrops norvegicus* **L.** [29], [30] and crab *Petrolisthes cinctipes* [31] has multiple paternity been reported. Freshwater shrimp species belonging to Malacostraca provide an excellent opportunity to study of the occurrence and frequency of multiple paternity because aquarium observations frequently detected multiple mating in freshwater ornamental shrimp species. The freshwater shrimp species *Caridina ensifera blue,* belonging to atyid genus *Caridina*, was discovered recently in Sulawesi, Indonesia [32]. This species is characterized by small body size (<2.5 cm), low fecundity and colourful appearance. Due to their colourful phenotypes, this species is favoured in freshwater aquaria [33]. However, nothing is known about its mating systems in nature. Therefore, studies on mating systems facilitate understanding of its reproductive strategies and how low fecundity species persist and evolve.

In this study, we developed four novel polymorphic microsatellites in *C. ensifera* and applied these markers to determine the occurrence of multiple paternity in 20 broods. This study allows addressing of important questions related to mating systems, and their importance in the persistence and evolution of this low fecundity species.

## Methods

### Ethics Statement

All handling of shrimps was conducted in accordance with the guidelines on the care and use of animals for scientific purposes set up by the Institutional Animal Care and Use Committee (IACUC) of the Temasek Life Sciences Laboratory, Singapore.

### Collection of females and DNA extraction

**Figure 1:**
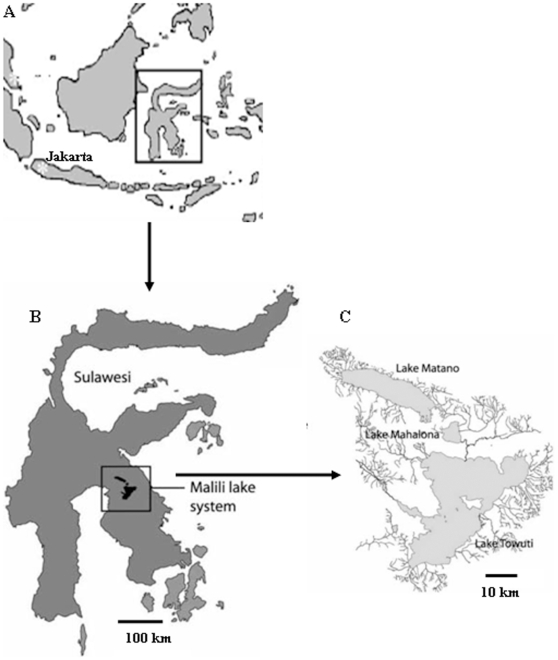
Sampling location of *C. ensifera blue* in Lake Matano in Sulawesi Island, Indonesia. A: Map of Indonesia; B: Map of Sulawesi and C: Map of Lake Mantano. The star showed the position of sampling *C. ensifera blue* individuals.

Twenty gravid females of *C. ensifera blue* were collected from the south part (02° 33.57′ S, 121° 25.19 E) of Matano Lake, Sulawesi, Indonesia (Fig. 1) in June 2008. All of the 20 live females were brought to Singapore and were raised in a laboratory at Temasek Life Sciences Laboratory. A small piece (2×2 mm^2^) of tail of each gravid female and all fertilized eggs/hatchlings attached to females were collected, immersed and stored in 95% ethanol. The number of fertilized eggs/hatchlings from each female was recorded. To examine whether the females store sperm, after collection of tissue samples and removing all fertilized eggs and hatchlings, all adult females were raised in a 0.25 m^3^ tank at 81 °F and pH 7.0. The shrimps were fed with HBH Crab & Lobster Bites (HBH). We examined whether the females bear fertilized eggs/hatchlings every week for nine months. DNA was isolated from the tissues of each gravid female and from fertilized eggs and hatchlings on 96-well PCR plates using the method that we developed previously [34].

### Developing and genotyping microsatellites

Microsatellites were isolated following the protocol described previously [35] with minor modifications [36]. Briefly, 450 ng genomic DNA was digested using 10 units of *Rsa I* (New England Biolabs) at 37°Cfor 2 h, followed by the ligation of the 21- and 25-mer adaptors [35]. Ligated DNA was PCR amplified using the 21-mer adaptors as primer in a 25 µL reaction consisting of 1× PCR buffer containing 50 mM KCl; 10 mM Tris-HCl (pH 8.3), and1.5 mM MgCl_2_, 400 nM of a 21-mer adaptor as primers, 200 µM dNTPs and one unit DNA polymerase (Finnzymes). The following PCR program was used: 94°C for 2 min, followed by 30 cycles of 94°C for 30 sec, 56°C for 30 sec and 72°C for 48 sec, and then a final extension at 72°C for 5 min. The PCR products were cleaned and concentrated using glassmilk (Gen 101). Biotinylated (CA)_10_ and (GA)_10_ (ProLigo) were used to perform hybridization reactions at 55°Cfor 30 min following a protocol described previously[35]. DNA enriched with CA-and GA-repeats was eluted in 30 µL distilled water at room temperature. DNA of 1 µL was amplified using the 21-mer adaptor as primer as described above. The resulting double-stranded products were cleaned using glassmilk (Gene 101). About 25 ng cleaned PCR products were ligated into 25 ng pGEM-T vector (Promega) and transformed into competent cell DH5-α (Stratagene). Inserts of white colonies were PCR amplified using M13 and M13 reverse primers. PCR was conducted on a PTC-100 thermocycler (BIO-RAD). The PCR program consisted of a denaturation at 94°Cfor 2 min followed by 35 cycles at 94°C for 30 s, 55°C for 30 s, and 72°C for 30 s, followed by a final extension at 72°C for 5 min. Inserts between 250–1200 bp were sequenced in both 5′ and 3′ directions using M13 and M13 primers, and BigDye chemicals on an ABI 3730xl DNA sequencer (Applied Biosystems). Forward and reverse sequences were assembled using SEQUENCHER (GeneCodes). Primers were designed in the flanking regions of each microsatellite using PRIMERSELECT (DNAstar). One primer of each pair was labelled with a fluorescent dye (6-Fam or Hex) to detect the PCR products using the ABI 3730xl sequencer (Applied Biosystems).

Genotyping of microsatellites was conducted using fluorescently labelled primers and an automated DNA sequencer ABI 3730xl (Applied Biosystems). Each microsatellite locus was amplified in 25 µl total volume containing 1× PCR buffer (Finnzymes) with 1.5 mM MgCl_2_, 200 nM of each primer, 100 µM dNTPs and one unit DNA polymerase (Finnzymes). PCR conditions were as follows: an initial denaturation step for 2 min at 94°C, followed by 35 cycles of denaturation (94°C for 30 s), annealing (55°C for 30 s) and extension (72°C for 30 s), and a final extension at 72°C for 5 min. PCR products were electrophoresed using the ABI 3730xl DNA sequencer, and allele sizes at each locus were determined against the size standard ROX-500 (Applied Biosystems). Data were analyzed using GeneMapper v3.7 (Applied Biosystems). Allele number, observed and expected heterozygosity, linkage equilibrium and Hardy-Weinberg equilibrium were analyzed using GDA [37].

### Analysis of parentage

Analysis of paternity was carried out by constructing a multilocus genotype for each embryo/hatchling, and then subtracting observed maternal alleles for each locus to obtain its paternally derived alleles. This analysis was conducted with the help of the GERUD software [38], as the software GERUD has been extensively used for parentage analysis in natural populations [28], [39], [40], [41], [42]. The occurrence of multiple paternity of a brood was unambiguously established by the occurrence of more than two paternal alleles across at least two loci, to allow for the possibility of mutation at one locus. For any brood where more than two paternal alleles were observed at only one locus, we used χ^2^ statistics to test whether the remaining three loci displayed evidence for significant deviations from expected Mendelian genotypic ratios. The null hypothesis for this test was that two alleles observed among a group of brood-mates were inherited from a single heterozygous father (i.e. with an expected ratio of the two alleles of 1∶1). Where multiple paternity was detected clearly, the program GERUD [38] was used to estimate the minimum number of males and to infer the most possible genotypes of males. To examine whether paternal contributions deviated significantly from equality in each brood, goodness-of-fit χ*^2^*-tests were applied. Because the program GERUD can only estimate the minimum number of males up to six individual, we repeated the analysis using another program COLONY [43], a likelihood-based program that provides the most likely paternity configuration and does not limit the number of fathers. In the analysis with COLONY, the error rate of genotyping was set to 0.025 as suggested by Wang [44]. The most probable genotypes of inferred fathers were used for further statistical analysis.

We performed an ordinal logistic regression analysis to assess whether the number of offspring and the increased number of alleles in offspring (as compared to the allele number in the mother and the father contributing most to the offspring in each brood) depend on the number of detected fathers in each brood, designating the number of offspring as the dependent variable. An ordinal scaling was chosen for the number of detected fathers since the paternity analysis with GERUD did not allow differentiating between six or more fathering males. For the data estimated by using COLONY, we performed a linear regression analysis to assess whether the number of offspring and the increased number of alleles in offspring depend on the number of inferred fathers in each brood, as the program COLONY was able to infer all possible sires in each brood. The analysis was carried out with the program JMP (SAS Institute). We also conducted a linear regression analysis to examine whether the genetic relatedness between the fathers and mothers was associated with the relative contribution of fathers to offspring. Genetic similarity (GS) between individuals *i* and *j* was estimated according to the formula given by Nei and Li [45]: GSij = 2*N*ij/(*N*i + *N*j), where *N*ij is the number of alleles common in individuals *I* and *j*, and *N*i and *N*j are the total number of allele in individuals *i* and *j*, respectively.

## Results

### Characterization of microsatellites

Microsatellites were identified from a partial genomic DNA library enriched with CA- and GA-repeats. Out of 48 clones sequenced, 15 contained microsatellites and enough flanking sequences in both 3′ and 5′ end. Primers were designed for 10 microsatellites. Four microsatellites (*BlueS02*, *BlueS03*, *BlueS04* and *BlueS07*) could be easily amplified and scored and showed polymorphism in 20 females. In the 20 mothers, the allele number ranged from 4 for the locus *BlueS03* to 25 for the locus *BlueS02* with an average of 13.5 alleles/locus (Table 1). Data analysis showed that the four loci were in linkage equilibrium and HWE. Paternity exclusion probability for all four loci combined was 99.80%.

**Table 1:** Characterization of four microsatellites from the genome of *Caridina ensifera blue*.

Locus (GenBank no)	Motif	Primer	Size (bp)	Allele No.	*Ho*	*He*
*BlueS02* GQ280906	(TAC)_17_	AGCGGACACCTGTAGATTACCT GAAGCCCCTAAATATTGGTTGTA	246	25	0.96	1.00
*BlueS03* GQ280907	(GACA)_6_	AGGTTTCGATTCCCCAAAGAGG GAAATGGCCAAGGGTTGTTCTG	398	4	0.52	0.75
*BlueS04* GQ280908	(ACAT)_20_	ATGTGGAAATACGGGGAATGTA CCCCGAAAATTTAATTAAGATGAT	215	20	0.96	0.90
*BlueS07* GQ280909	(AG)_32_	CTCATCAGGTTGACGGAGAGAG CCAAACTGCATGAATCCAGACT	350	5	0.70	0.95

*Ho*: observed heterozygosity; *He*: expected heterozygosity.

### Parentage

**Figure 2:**
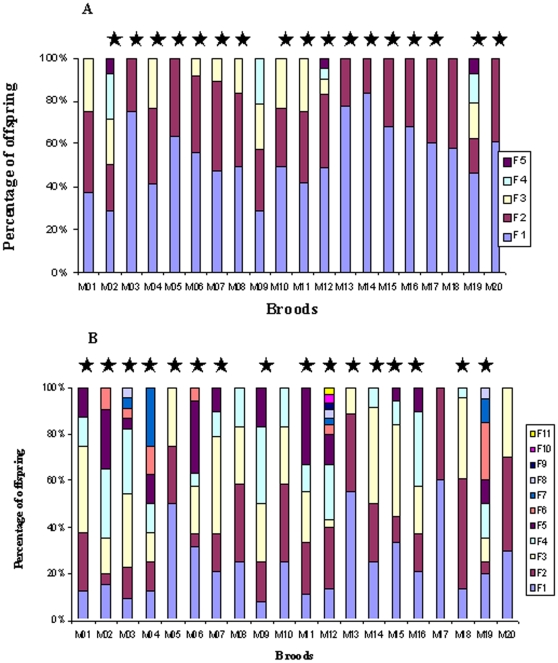
Relative contribution of fathers to broods sired by multiple fathers. A: Data estimated using software GERUD and B: Data estimated using the program COLONY. F1-F11: Fathers 1–11 in broods. The star above a bar indicates paternal contributions deviated significantly from equality (goodness-of-fit χ*^2^*-tests, *P*<0.05) in a brood.

Twenty females with embryos and hatchlings attached to their pleopods were collected. The average brood size was 15.65±1.65, ranging from 5 to 30 (Table 2). The developing stage of all offspring within each brood was identical, but was obviously different among broods (Table 2). We found evidence of multiple paternity in all 20 broods (Table 2) using two different programs GERUD and COLONY. Each embryo and hatchling contained at least one allele from the mother, and χ^2^ tests confirmed that inheritance of these alleles did not vary from 1∶1 Mendelian inheritance within each brood (df = 1, *P* = 0.0575∼0.80). With the data estimated using GERUD, we found that he female holding the embryos and hatchlings was exclusively the mother of these offspring. Six broods had a minimum of three different fathers and three had five different fathers (Table 2). In most broods, we could not confidently assign specific sires to individual progeny due to extensive allele sharing between fathers and mothers. Several different solutions of male genotypes with similar relative contributions to the offspring were determined by GERUD. In such cases, we selected the most potential fathers according to the ranking based on the Mendelian segregation of alleles and the allele frequencies in the population. In 17 of the 20 broods, paternal contributions deviated significantly from equality (goodness-of-fit χ*^2^*-tests, *P* = 0.0001∼0.0415; Fig. 2A). Only in three broods: M1 (*P* = 0.14), M9 (*P* = 0.47) and M18 (*P* = 0.11), sires contributed almost evenly to the offspring. We did not find the same genotypes at all four loci among the 58 inferred fathers. Similar results were obtained by using the program COLONY. Multiple paternity was found in each of the 20 broods. However, the number of sires in each brood except the brood M17 was larger than that estimated using software GERUD. A total of 105 sires were inferred in all 20 broods. The sire number in the 20 broods ranged from 2 for brood M17 to 11 for blood M12 (Table 2) with an average of 5.25±0.49 sires/brood. In 16 of the 20 broods, paternal contributions deviated significantly from equality (goodness-of-fit χ*^2^*-tests, *P* = 0.010 - 0.0012; Fig. 2B). Only in four broods: M8 (*P* = 0.14), M10 (*P* = 0.14), M17 (*P* = 0.06) and M20 (*P* = 0.36), sires contributed almost evenly to the offspring.

**Table 2:** Sample size, offspring stage and number of sires in 20 broods of *Caridina ensifera blue* inferred using GERUD and COLONY software*.

Dam'ID	No. of offspring	Offspring stage	Number of sires **
M01	8	Hatchling	3/5
M02	20	Hatchling	5/6
M03	22	Fertilized egg	2/8
M04	16	Hatchling	3/7
M05	8	Fertilized egg	2/3
M06	20	Hatchling	3/7
M07	19	Hatchling	3/5
M08	12	Hatchling	3/4
M09	12	Hatchling	4/5
M10	19	Fertilized egg	3/5
M11	9	Fertilized egg	3/5
M12	30	Hatchling	5/11
M13	9	Fertilized egg	2/3
M14	12	Hatchling	2/4
M15	18	Hatchling	2/5
M16	19	Fertilized egg	2/5
M17	5	Hatchling	2/2
M18	25	Hatchling	2/4
M19	20	Hatchling	5/8
M20	10	Hatchling	2/3
Mean (Se)	15.65 (1.65)		2.95 (0.30)/5.25 (0.49)

*: GERUD infers the minimum number of offspring whereas COLONY infers the number of most likely offspring.

**: The number before/was inferred by using GERUD and after/was inferred by using COLONY software. Se: standard error.

**Figure 3:**
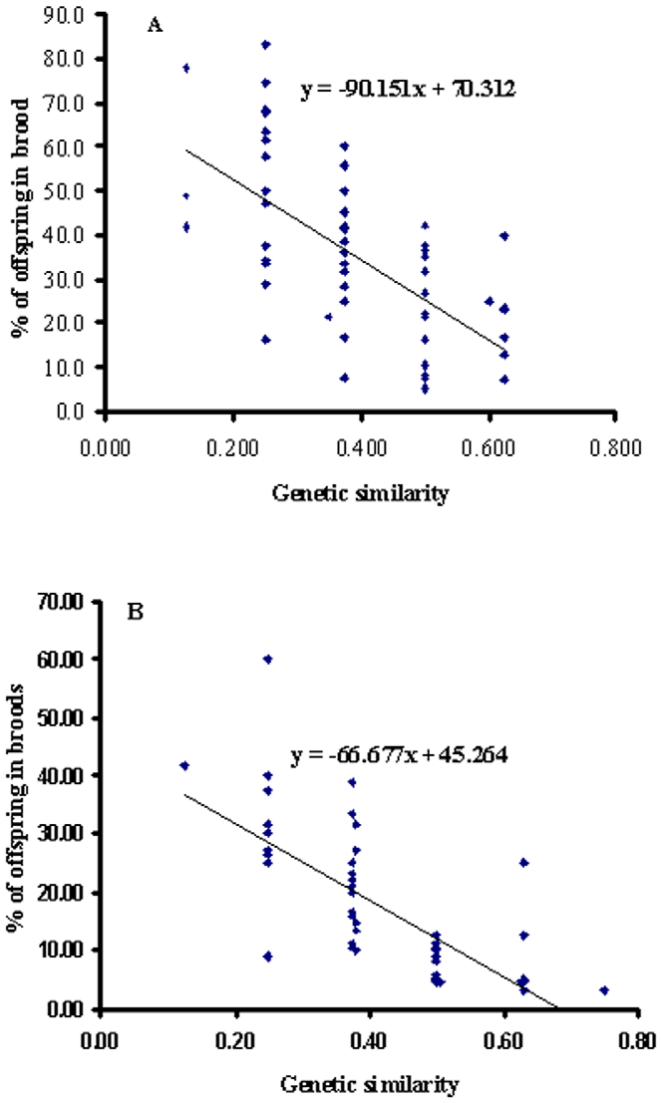
The percentage of offspring sired by a father plotted against the genetic similarity between the father and mother. The equation of the linear regression is shown on the figure. A: Analysis based on data estimated using software GERUD and B: Analysis based on data estimated using the program COLONY.

According to data estimated using the program GERUD, the number of offspring in each brood was not significantly associated with the number of fathers observed (χ*^2^* = 2.18, df = 1, *P* = 0.14), whereas the allele number in offspring was significantly associated with the number of fathers in broods (χ*^2^* = 3.86, df = 1, *P* = 0.047). Genetic similarity between fathers and mothers was negatively (*r^2^* = 0.40, df = 1, *P* = 2.58E^−16^) associated with the percentage of offspring sired by the father (Fig. 3A), indicating there was an inverse regression between reproductive success of sires and their relatedness to mothers. Based on the data estimated with software COLONY, the number of offspring in each brood was significantly associated with the number of fathers observed (*r^2^* = 0.58, df = 1, *P* = 9.90E^−05^). Similarly the allele number in offspring in each brood was significantly associated with the number of fathers observed (*r^2^* = 0.51, df = 1, *P* = 0.0004). Genetic similarity between fathers and mothers was negatively (*r^2^* = 0.65, df = 1, *P* = 9.1E^−25^) associated with the percentage of offspring sired by the father (Fig. 3B).

### Storage of sperm by females

After removing fertilized eggs and hatchlings from the 20 adult females, we put the adult females in a tank without any males, and monitored whether the females produce eggs and held fertilized eggs every week for nine months. We did not detect any females holding fertilized eggs or hatchling, but saw eggs, suggesting no sperm was stored to fertilize eggs in the female after the fertilization of the first batch of eggs.

## Discussion

Although there are over 22,000 species in Malacostraca [19], little is know about parentage in populations. The present study provides the first insights into the occurrence and frequency of multiple paternity in a freshwater shrimp species *C. ensifera blue,* a member in Malacostraca.

The average brood size in *C. ensifera blue* was only 15.65±1.65. Similar low number of offspring has been reported in other endemic Atyid shrimp species: *C. longidigita* [46] and *C. spongicola* [47] in Sulawesi. The brood size of *C. ensifera blue* is much smaller than that of most freshwater shrimp species [48], indicating that low fecundity is a reproductive characteristic of the endemic Atyid shrimp species in Sulawesi. The low number of offspring may be related to the small body size of *C. ensifera blue*. Low fecundity could lead this species more vulnerable to catching pressure and climate change. In shrimp species, usually females take care of the offspring by carrying them until hatching. In this study, we found all offspring attached to the females were the offspring of the mothers. This extended mother care might be important for the survival of offspring. High survival rate of offspring is especially important for low fecundity species for persistence and adaption to environmental change because any loss of offspring could reduce the potential for retaining evolutionary potential.

In this study, multiple paternity was detected in all 20 broods using two different methods as implemented in programs GERUD [38] and COLONY [43], respectively. The frequent occurrence of multiple paternity in *C. ensifera blue* (100%), crayfish species *Orconectes placidus* (40%) [27], the American lobster, *Homarus americanus* (13% of 108 females) [49], the Norway lobster, *Nephrops norvegicus* (54.6% of 11 broods) [30], the porcelain crab, *Petrolisthes cinctipes* (80% of 10 brooders) [31], suggests multiple paternity is common in Malacostraca.

The high degree of multiple paternity suggests that sperm must be mixed within the female's reproductive tract. During mating, the male deposits the sperms into the female before the eggs are passed from the ovaries and into the undercarriage. As the eggs are passed down into the undercarriage, they become fertilized by the previously deposited sperm. Although in *C. ensifera blue*, sperms were stored in the female reproductive organ, after fertilization and hatching, no sperms were stored, indicating that in *C. ensifera blue*, sperms were only used for fertilization of eggs of one spawning, and were lost after ecdysis. This short time storage of sperms could enable postcopulatory selection of sperm and/or sperm competition [50], [51]. Using two different methods with software GERUD and COLONY to infer paternity, we found skewed contribution of different fathers to offspring and lower contribution of genetically similar males to the offspring in most broods, suggesting that sperm competition occurred and/or pre- and postcopulatory female choice happened. Pre-copulatory female choice might be possible, as females may mate more than once with preferred males [52]. The postcopulatory female choice may be accomplished in a number of ways, such as by dumping unwanted sperm, as observed in *L. scabra* [53], by sperm digestion in the bursa copulatrix [54], or by sperm sorting and differential use within the reproductive tract [55]. Relatively higher contribution of genetically distinctly related fathers to offspring may be important to optimize genetic variation in offspring and to avoid inbreeding [14], [15]. The postcopulatory female choice that produces a genetically diverse offspring generation could obviously increase the genetic diversity that is maintained at equilibrium [56], thus feeding back and reinforcing the evolution of the species. It is also likely that females may select the sperm from genetically most compatible males for fertilization. In such a case, offspring fitness depends on an interaction between the maternal and paternal haplotypes/alleles [13]. However, in this study, it is impossible to differentiate sperm competition and postcopulatory female choice. To fully understand the occurrence of sperm competition and/or cryptic female choice in this species, experiments must be designed that track the sperm of individual male inside the female, as well as in the resulting offspring. This could be accomplished by using artificial fertilization of mixed sperms from different males and parentage analysis using genetic markers.

The conditions that determine and influence the frequency of multiple paternity are largely unknown. Previous studies suggested that when females incur the bulk of the energetic investment of reproduction and receive no postcopulatory investment from males, multiple mating is favoured by females [12]. This seems to be also the case of *C. ensifera blue* shrimp, as after mating, only the females take care of the offspring. Similar mating behaviour of females has been reported in crayfish species *Orconectes placidus* [27]. Polyandry may also arise from parent-offspring conflict over parent investment or during group courtship when several males try to copulate with one female during a mating aggregation [57]. This kind of behaviour has been reported in several animal species [58], [59], [60], but never has been in shrimp species. The frequency of multiple paternity may be related to population density [61], [62], [63], site fidelity and catching pressure. Our next step is to investigate how frequency of multiple paternity varies with the level of exploitation.

We have noticed that although using two different approaches implemented in GERUD and COLONY, the general conclusion about multiple paternity, and inverse correlation between reproductive success of sires and their relatedness to mothers was the same, the most probable number of sires given by COLONY is higher than the minimum number given by GERUD. Similar results were reported in several other studies on paternity [64], [65]. Although some studies suggested that GERUD outperformed over COLONY [66], [67], and vice versa [65], [68]. We would like to suggest using both software for parentage analysis, as previous studies demonstrated that the relative performances of software for parentage analysis depend on brood size, the true number of sires, the polymorphisms of DNA markers and the distribution of parental contributions between sires [65]. Using both approaches may give a comprehensive view on multiple paternity.

### Conclusion

We analyzed the occurrence and frequency of multiple paternity of a new freshwater shrimp species *C. ensifera blue* using polymorphic microsatellite markers. Our result in combination with recent reports of crayfish, crab and lobster suggests that multiple paternity is common in Malacostraca. The skewed contribution of different fathers to offspring, and lower contribution of genetically similar males to the offspring suggest that sperm competition occurred and/or pre- and postcopulatory female choice happened to optimize genetic variation in offspring and to avoid inbreeding. High prevalence of multiple paternity may impact male reproductive success and possibly contribute to increase female and offspring fitness, which is important for low fecundity shrimp species for persistence and evolution.
